# Diseases associated with leaky hemichannels

**DOI:** 10.3389/fncel.2015.00267

**Published:** 2015-07-27

**Authors:** Mauricio A. Retamal, Edison P. Reyes, Isaac E. García, Bernardo Pinto, Agustín D. Martínez, Carlos González

**Affiliations:** ^1^Centro de Fisiología Celular e Integrativa, Facultad de Medicina, Clínica Alemana Universidad del DesarrolloSantiago, Chile; ^2^Centro de Investigación Biomédica, Universidad Autónoma de ChileSantiago, Chile; ^3^Centro Interdisciplinario de Neurociencia de Valparaíso, Instituto de Neurociencia, Facultad de Ciencias, Universidad de ValparaísoValparaíso, Chile

**Keywords:** connexins, leaky hemichannels, mutations, gap junction channels, cell death, disease

## Abstract

Hemichannels (HCs) and gap junction channels (GJCs) formed by protein subunits called connexins (Cxs) are major pathways for intercellular communication. While HCs connect the intracellular compartment with the extracellular milieu, GJCs allow the interchange of molecules between cytoplasm of two contacting cells. Under physiological conditions, HCs are mostly closed, but they can open under certain stimuli allowing the release of autocrine and paracrine molecules. Moreover, some pathological conditions, like ischemia or other inflammation conditions, significantly increase HCs activity. In addition, some mutations in Cx genes associated with human diseases, such as deafness or cataracts, lead to the formation of more active HCs or “leaky HCs.” In this article we will revise cellular and molecular mechanisms underlying the appearance of leaky HCs, and the consequences of their expression in different cellular systems and animal models, in seeking a common pattern or pathological mechanism of disease.

## Introduction

**FIGURE 1:**
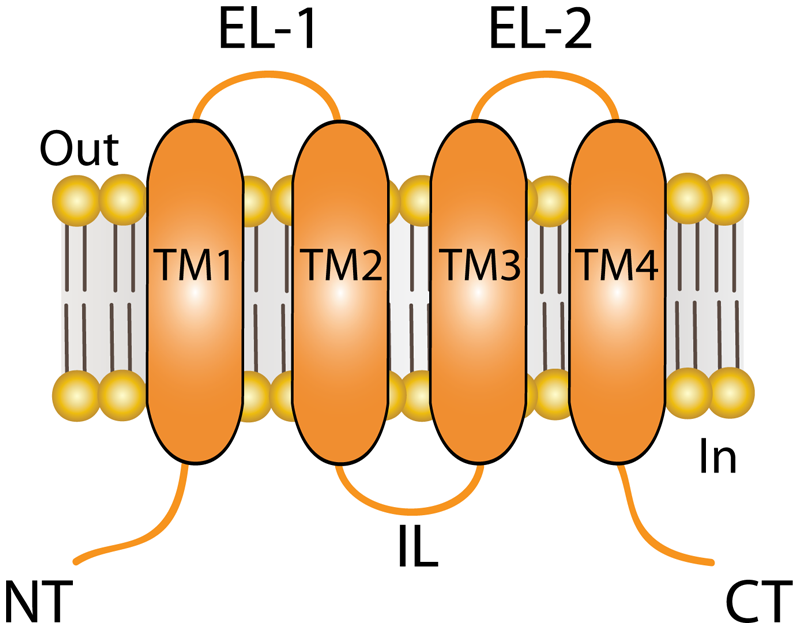
**Topology of connexin (Cx) at the plasma membrane.** Cartoon depicting the plasma membrane topology shared by all Cx isoforms, which includes four transmembrane (TM) segments that are connected by two extracellular loops (ELs) and one intracellular loop (IL). The amino terminal (NT) and carboxi terminal (CT) segments of each hemichannel face the cytoplasm. The length of the NT and CT segments is not intended to represent any particular Cx isoform.

**FIGURE 2:**
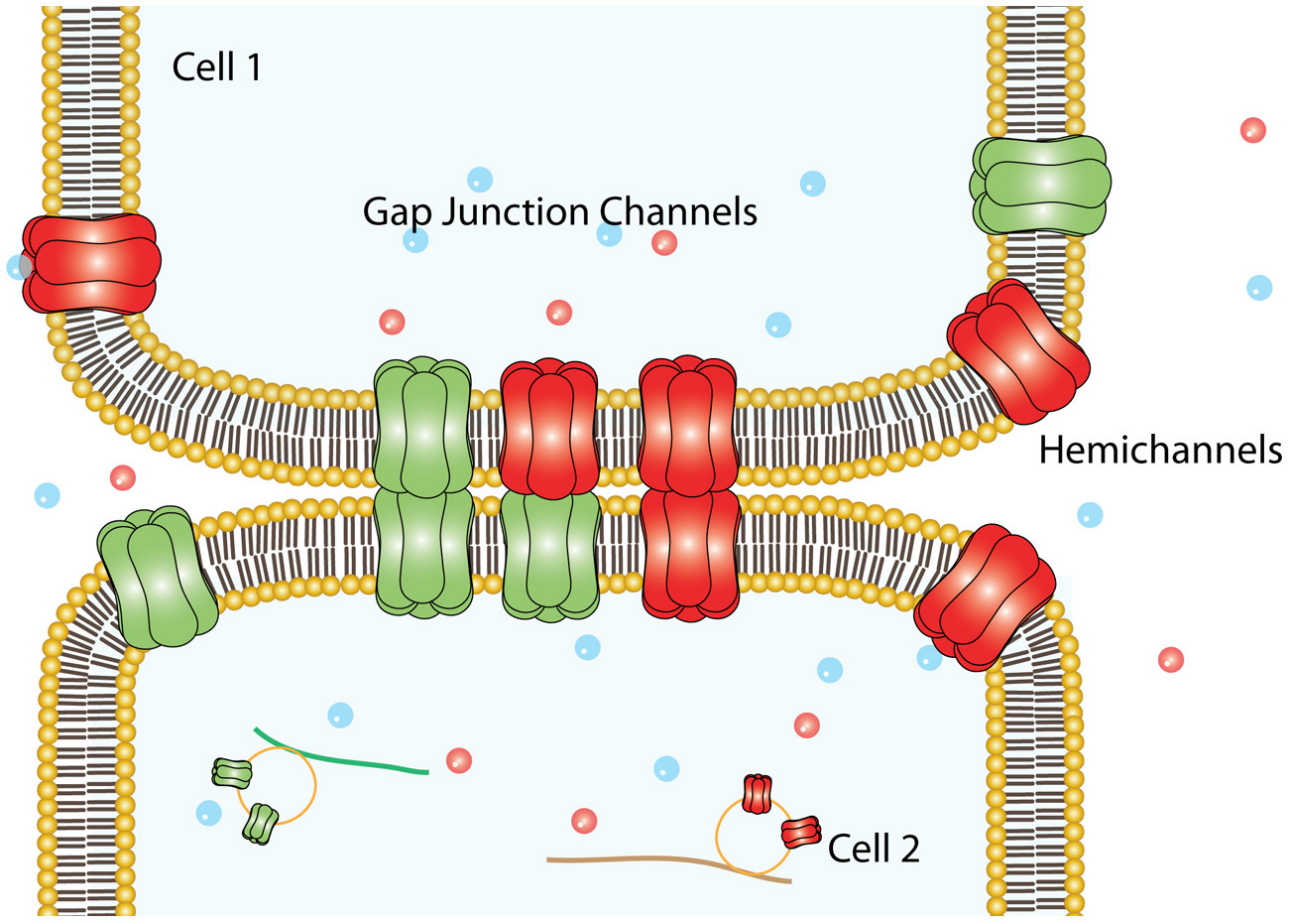
**Plasma membrane arrangements of Cxs.** Six Cxs oligomerize to form a HC that traveled to the non-appositional plasma membrane to form free HCs, which provide an auto/paracrine communication pathway between the cell and the extracellular milieu. Alternatively, can dock others HCs provided by an adjacent cell (appositional plasma membrane) to form intercellular aqueous pore named gap junction channels.

Connexins (Cxs) are a family of transmembrane (TM) proteins formed by 21 members (Eiberger et al., 2001; Söhl and Willecke, 2004) named according to their predicted molecular weight (i.e., Cx43 has ∼43 kDa). Cxs are expressed in almost every cell type in the human body (Bruzzone et al., 1996). However, there are some differences. Thus, for example, there are Cxs widely expressed such as Cx43, which is found in the brain, kidneys, heart and reproductive organs, among others (Beyer et al., 1987, 1989; Sáez et al., 2003), or restricted to myelin-forming glial cells, as in the case of Cx29 (Söhl et al., 2001). Cxs form two types of channels; hemichannels (HCs) and gap junction channels (GJCs). HCs are formed by the oligomerization of six Cxs monomers and travel in vesicles to the plasma membrane (Vinken et al., 2006). The Cx topology in cell membrane is depicted in **Figure 1** and includes four TM segments (TM1-4), which are connected through two extracellular loops (EL1-EL2) and one intracellular loop (IL); and the N-terminal (NT) and C-terminal (CT) segments oriented to the cytosol (Kumar and Gilula, 1996). HCs can form GJC in the appositional membranes of contacting cells or stay as “free” HCs anywhere on the plasma membrane (**Figure 2**). Free HCs are mostly closed under physiological conditions (Contreras et al., 2003), that is because they have low open probability (OP) due to one or more of the following mechanisms: (i) a blockage by extracellular Ca^2+^ and Mg^2+^ in the mM range, (ii) a negative membrane potential that closes most Cx HCs and (iii) posttranslational modification (i.e., phosphorylation) of some Cxs (Contreras et al., 2003; Gómez-Hernández et al., 2003; Johnstone et al., 2012). However, HCs can open under physiological conditions allowing communication between extracellular and intracellular space (Sáez et al., 2010). On the other hand, GJCs are formed in the appositional membrane by the serial docking of two complementary HCs, each one in the respective neighboring cell membrane (**Figure 2**). GJCs allow the intercellular exchange of ions and molecules such as glucose and amino acids between contacting cells (Payton et al., 1969; Goldberg et al., 2004; Ek-Vitorin and Burt, 2013). Because of these properties, Cx based channels have been associated with different cellular processes such as cellular communication and tissue coordination (Sáez et al., 2010).

## Role of HCs in Physiological Conditions

HCs have an estimated pore diameter ranging from 12 to 15 Å in its narrowest part (Oh et al., 1997; Gong and Nicholson, 2001; Rackauskas et al., 2010). The crystal structure of Cx26 channels shows that the NT is inside the pore, a factor that restricts the pore diameter (Maeda et al., 2009). However, recent refinements of this structure using molecular dynamic methods suggest that the pore diameter could be a little smaller (Kwon et al., 2011). Much experimental evidence shows that opening of HCs activates pathways linked to the release or uptake of paracrine and autocrine molecules such as: ATP (Anselmi et al., 2008 (Cx26); Svenningsen et al., 2013 (Cx30); Nualart-Marti et al., 2013 (Cx32); Schock et al., 2008 (Cx36); Stout et al., 2002 (Cx43)), glutamate (Takeuchi et al., 2006 (Cx32); Ye et al., 2003 (Cx43)), PGE_2_ (Cherian et al., 2005 (Cx43)), NAD^+^ (Bruzzone et al., 2001 (Cx43)) and glutathione (Rana and Dringen, 2007 (Cx43)). HCs may also mediate uptake of glucose as well as extracellular ions. (Retamal et al., 2007 (Cx43); Schalper et al., 2010 (Cx43); Sánchez et al., 2010 (Cx26); Fiori et al., 2012 (Cx26)). Research about HC permeability has been focused mostly on homomeric HCs made by Cx26, Cx32 and Cx43. However, most cell types express more than one Cx isoform, opening the possibility for the formation of heteromeric channels that would present new permeability properties (Beyer et al., 2001; Martinez et al., 2002). For example, it is known that heteromeric HCs formed by Cx26/32 (1:1 ratio) exhibits decreased permeability to (1,4,5)-IP_3_ compared to the respective homomeric types formed by Cx26 or Cx32 (Ayad et al., 2006). Additionally, information about the *in vivo* release of molecules through HCs is currently very limited. However, data available suggest that HCs are somehow involved in different physiological processes, such as the control of monocyte adhesion in mice (Wong et al., 2006), neurotransmitter release from astrocytes in the basolateral amygdala (Stehberg et al., 2012), Ca^2+^ signaling in adult ventricular myocytes (Li et al., 2012), sensory neuron activity (Retamal et al., 2014b), and bone cell physiology and pathology (Plotkin, 2014). Moreover, HCs may also participate in the ATP release from astrocytes to regulate basal glutamatergic synaptic transmission (Chever et al., 2014), in the control of colonic transit (McClain et al., 2014), in wound healing (Takada et al., 2014), in renal function (Sipos et al., 2009), ion flux in lens cells (Beyer and Berthoud, 2014; Mandal et al., 2015) and in the visual processing of the retina (Kamermans et al., 2001). The signaling and molecular mechanisms that control the opening of HCs under physiological conditions are poorly understood. But, at least for Cx43 HCs, one possible mechanism involves interactions between the CT and some regions of the IL (Ponsaerts et al., 2010, 2012).

The presence and functional state of HCs in the plasma membrane have been determined through several techniques, including, dye uptake of fluorescent molecules, release of substances such as ATP, electrophysiology, biotinylation, immunolocalization (Schalper et al., 2008; Wang et al., 2013a; García et al., 2015). Because the existence of other non-selective channels with big pores, like Pannexin channels, which share several characteristics with Cx HCs, there are some criterions that need to be considered in order to demonstrate unique functional properties of Cx HCs (Sáez and Leybaert, 2014). Among these criterions are: (i) cell expression of at least one Cx isoform at the plasma membrane, (ii) the ability of the cells to incorporate/release fluorescent dyes, and /or (iii) to show currents with conductance and/or properties associated to Cx HCs, (iv) the abolishment of Cx-HCs function using classical pharmacology (La^3+^, mefloquine, carbenoxolone) or Cx mimetic peptides (Gap 26, 27); and finally (v) to demonstrate that blocking Cx HCs exerts physiological responses.

## Leaky HCs

Paul et al. (1991) provided the first evidence linking HCs with cell death, they observed that overexpression of Cx46 in *Xenopus laevis* oocytes, induces depolarization and lysis of oocytes 24 h after mRNA injection. Interestingly, cell death did not occur when other Cxs, like Cx32 or Cx43 were overexpressed. Later on, it was demonstrated that human Cx43 does not form functional HCs in *Xenopus* oocytes (Hoang et al., 2010), suggesting that formation of functional HCs depends on both Cx isoform and cell type. On the other hand, several works have shown that many pathological conditions produce uncontrolled and massive HC opening (from now called leaky HCs), which may adversely affect cellular homeostasis and induce cell death (Sáez et al., 2010). For example, it was reported that infection of the gastrointestinal tract increases Cx43 HC activity in colonocytes, which was correlated with an increase of water mobilization and appearance of diarrhea, which can be reverted by down-regulating gut expression level of Cx43 (Guttman et al., 2010). In addition, natural occurring Cx mutations have been associated with different human genetic diseases such as cataract, skin diseases, deafness, X-linked Charcot–Marie–Tooth disease, and oculodentodigital dysplasia (ODDD; **Table 1**). Some Cx mutations in these diseases produce leaky HCs when expressed in heterologous expression systems. Here, we will review the current knowledge about these leaky HCs and their possible pathologic mechanisms of disease.

**Table 1:** Connexin (Cx) mutations associated to leaky HCs.

	Cx	Mutation	Localization	Type of disease	Reference
Skin and inner ear	Cx26	G12R	NT	Keratitis-ichthyosis-deafness (KID) syndrome	Lee et al. (2009)
		N14K	NT	Keratitis-ichthyosis-deafness syndrome	Lee et al. (2009)
		N14Y	NT	Keratitis-ichthyosis-deafness syndrome	García et al. (2015)
		A40V	TM1/EL1	Keratitis-ichthyosis-deafness syndrome	Sanchez et al. (2014)
		G45E	TM1/EL1	Keratitis-ichthyosis-deafness syndrome	Stong et al. (2006), Gerido et al. (2007)
		D50N	EL1	Keratitis-ichthyosis-deafness syndrome	Lee et al. (2009)
		D50A	EL1	Keratitis-ichthyosis-deafness syndrome	Mhaske et al. (2013)
		A88V	TM2	Keratitis-ichthyosis-deafness syndrome	Mhaske et al. (2013)
Skin	Cx30	G11R	NT	Hidrotic ectodermal dysplasia	Essenfelder et al. (2004)
		A88V	TM2	Hidrotic ectodermal dysplasia	Essenfelder et al. (2004)
	Cx31	R42P	TM1/EL1	Erythrokeratodermia variabilis	Chi et al. (2012)
	CX43	G8V	NT	Keratoderma-hypotrichosis-leukonychia totalis syndrome	Wang et al. (2015)
Lens	Cx46	G2D	NT	Nuclear pulverulent and posterior polar cataracts	Yao et al. (2011)
		G143R	IL	Coppock cataracts	Ren et al. (2013)
	Cx50	V44A	TM1/EL1	Suture-sparing nuclear cataracts	Zhu et al. (2014)
		G46V	E1	Cataract	Tong et al. (2011)
Nervous system	Cx32	F235C	CT	Charcot–Marie–Tooth disease	Liang et al. (2005)
		S85C	TM2	Charcot–Marie–Tooth disease	Abrams et al. (2002)
	Cx43	G60S	EL1	Oculodentodigital dysplasia	Kozoriz et al. (2013)
		G138R	IL	Oculodentodigital dysplasia	Dobrowolski et al. (2008)
Heart	Cx40	G38D	TM1	Chronic atrial fibrillation	Patel et al. (2014)
		V85I	TM2	Atrial fibrillation	Sun et al. (2014)
		L211I	TM4	Atrial fibrillation	Sun et al. (2014)
	Cx43	I31M	TM1	Spontaneous arrhythmias	Dobrowolski et al. (2007)
		G138R	IL	Spontaneous arrhythmias	Dobrowolski et al. (2007)
		G143S	IL	Spontaneous arrhythmias	Dobrowolski et al. (2007)

*Leaky HCs can result from single point mutations. Mutations in different Cxs can lead the development of different diseases with hallmark characteristics. Note that most mutations leading to formation of leaky HCs are located in the N- terminus (NT), the extracellular loop 1 (EL1) and transmembrane domains 1 and 2 (TM1 and TM2). In a lesser extent, some mutations are found in the intracellular loop (IL) or the transmembrane domain 3 and 4 (TM3 and TM4). In terms of leaky HCs, mutations in C-terminal are rarer, probably because the first half of the protein including (NT, TM1, EL1, TM2 and IL) is much more important in terms of pore formation, permeability and voltage gating.*

### Cataract

**FIGURE 3:**
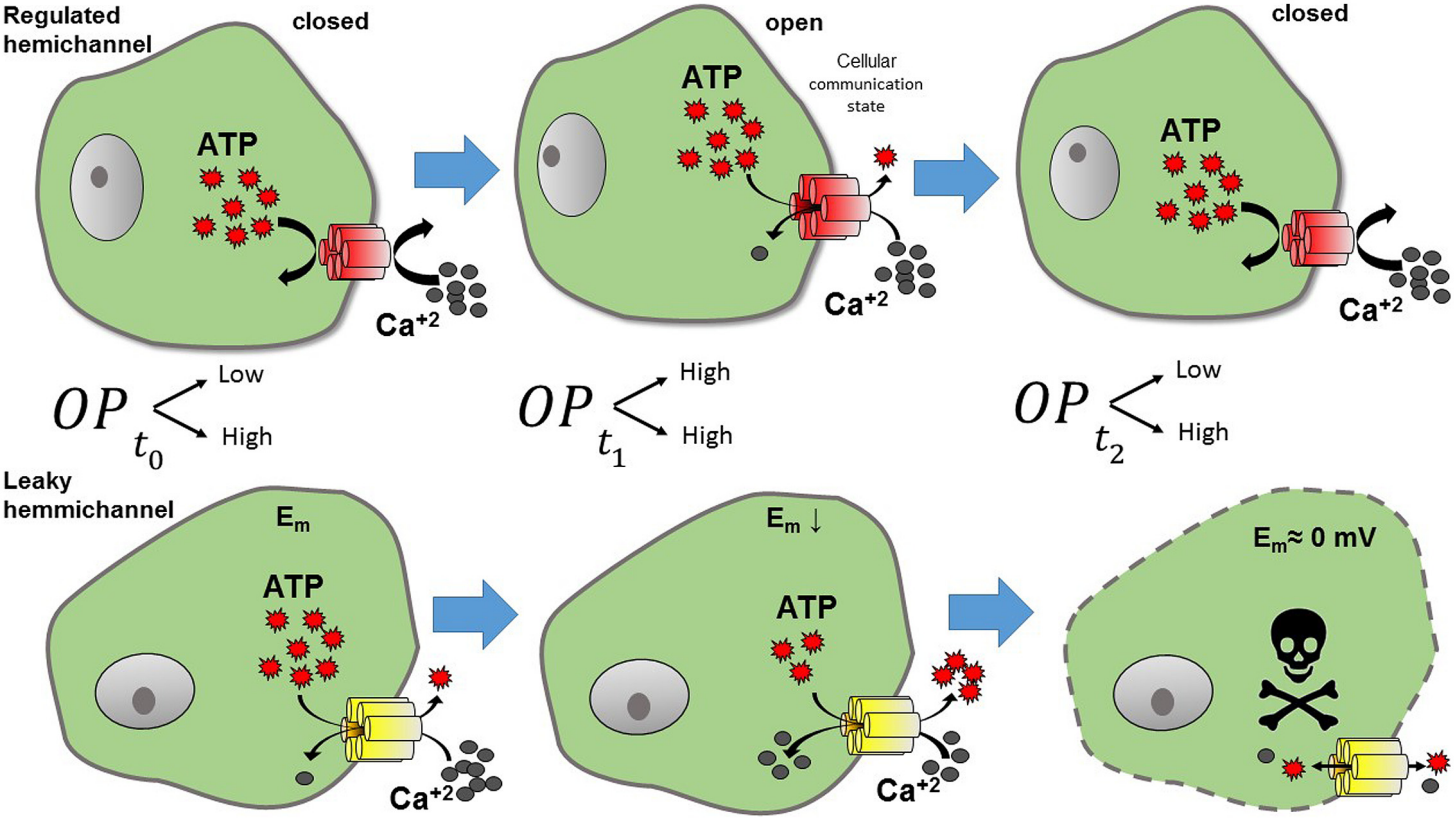
**Representation of the effects of leaky HC.** Under normal conditions **(upper panel)** HCs present a low open probability (OP). Thus, when HCs are normally closed (*t*_0_, low OP), no exchange with the extracellular milieu is observed. However, when HCs open (*t*_1_, higher OP), molecules such as ATP and Ca^2+^ can flow through them. Calcium may activate intracellular pathways, and ATP released from the cell, can act as a paracrine -or autocrine- signal, hence, the cell is at a communicating state. In contrast leaky HCs **(lower panel)** maintain a high OP, producing a continuous flow out and into the cell. Leaky HCs exchange continuously, resulting in the reduction of cell membrane potential and later cell death (*t*_2_).

Lens cells express Cx43, Cx46, and Cx50 (Beyer and Berthoud, 2014). Until now, Cx43 mutations have not been associated with cataract formation; however, the lack or malfunctioning of Cxs 46 and 50 has been associated with the development of different types of cataracts (Beyer and Berthoud, 2014). Accordingly, the expression of Cx50G46V mutant in *Xenopus* oocytes induces large HC currents at physiological extracellular Ca^2+^ concentration (Tong et al., 2011). In addition, this mutant promotes cell death when expressed in HeLa cells, which was prevented by the increase of the extracellular Ca^2+^ concentration (Minogue et al., 2009). Similarly, Cx50V44A mutant also induces HeLa cell death, which was reduced by HC blockers (Zhu et al., 2014). The aforementioned data suggest that some Cx50 mutations located closely to the TM1 and EL1 border result in formation of leaky HCs, which cause cataracts when expressed in humans. Additionally, different Cx46 mutations have been associated with leaky HCs. For example, mutant Cx46G143R (located in the IL) leads to the appearance of Coppock cataracts (Ren et al., 2013), and mutant Cx46G2D (located the NT) has been linked to formation of nuclear pulverulent and posterior polar cataracts (Yao et al., 2011). Lower plasma membrane expression of these mutants was enough to promote cell death when expressed in HeLa cells (Ren et al., 2013). This suggests that mutation Cx46G143R induces an important increase in the HC activity, possibly by modifying the interaction between the CT and IL, which is associated with HC opening (Ren et al., 2013). A possible explanation for the pathological mechanism of leaky Cx46 HCs is that the opening of these channels produces an excessive flow of Ca^2+^ through the plasma membrane (Ebihara et al., 2014; Mandal et al., 2015), which should perturb the normal ionic balance of lens cells (**Figure 3**).

### Skin Diseases and Deafness

Several Cx types such as Cx26, Cx30, Cx30.3, Cx31.1, Cx37, and Cx43 are differentially expressed in the skin (Scott et al., 2012). On the other hand, while in the inner ear the sensory hair cells do not express Cxs, several Cxs (Cxs 26, 29, 30, 31, 43) are expressed in supporting epithelial cells of the organ of Corti, stria vascularis and in the interstitial cellular network that compose the wall of the scala media (Martínez et al., 2009). However, until now, only mutations in Cx26 gene are associated to syndromic (deafness plus skin disease) and non-syndromic deafness (Hoang Dinh et al., 2009; Martínez et al., 2009). Currently it is known that several missense point mutation in Cx26 – G12R, N14K, N14Y, A40V, G45E, D50N, D50A and A88V do form leaky HCs and induce both skin and hearing disorders, which together are known as keratitis-ichthyosis-deafness (KID) syndrome (Stong et al., 2006; Gerido et al., 2007; Lee et al., 2009; García et al., 2013; Mhaske et al., 2013; Meigh et al., 2014; Sanchez et al., 2014). Interestingly, García et al. (2015) showed that the mutant Cx26S17F presents decreased HC activity when expressed alone in *Xenopus* oocytes, but when is co-expressed with Cx43 [which does not form functional HCs in *Xenopus* oocytes (Hansen et al., 2014)], a large HC current is then evident (García et al., 2015). Because of these leaky HCs, HeLa cells expressing Cx26S17F and Cx43 showed almost twice the basal intracellular Ca^2+^ concentration (García et al., 2015). These results could explain the resulting KID syndrome of the mutant S17F, since in the human skin Cx26 and Cx43 are co-expressed in keratinocytes of the stratum basal (Wang et al., 2009). In addition, certain mutations located in the EL1 also produce leaky HCs, such as D50N, that change the Ca^2+^ control over HC activity through the modification of a salt bridge between D50 and K61, which is important for HC closure induced by extracellular Ca^2+^ (Lopez et al., 2013; Sanchez et al., 2013). Consistently, a similar mutation (Cx26D50A) also induces leaky HC and produce KID syndrome (Mhaske et al., 2013). On the other hand, mutant Cx26A40V, located in the TM1/EL1 border, increases HC activity by decreasing sensitivity to pH and divalent cations (Sanchez et al., 2014). However, other mutation in the same residue (A40G) produced non-functional HCs (Jara et al., 2012) and severe deafness without skin disease, suggesting that this residue is positioned in a pivotal region for HC gating control. Another mutant associated with KID syndrome, Cx26G45E (Griffith et al., 2006) also induces large HC currents or dye uptake that leads to cell death when expressed in *Xenopus* oocytes or HEK293 cells (Stong et al., 2006; Gerido et al., 2007). The phenotype induced by the Cx26G45E mutant could be the result of a lack of Ca^2+^ control (Zhang and Hao, 2013), reflected by enhanced Ca^2+^ permeability in cells expressing this mutant (Sanchez et al., 2014). In a mouse model, Cx26G45E induces a reduction of cell viability, hyperkeratosis, scaling, skinfolds, and hair loss (Mese et al., 2011). These data suggest that leaky Cx26 HCs can induce skin problems and deafness, probably due to misregulation of intracellular Ca^2+^ homeostasis and ATP release (García et al., 2015) (**Figure 3**); both conditions could affect many cellular processes, such as abnormal keratinocyte proliferation and cell death. Although, most mutations in Cx26 associated with deafness produce lack of function GJCs, only syndromic mutations produce gain of HC activity (Martínez et al., 2009; García et al., 2013). However, since syndromic mutations also affect GJCs, the pathogenic mechanism of disease may involve deregulation of both types of channels, which is a condition that would worsen the cellular homeostasis (García et al., 2015).

Mutations in Cx30 have been also linked to skin disease. Thus, mutants of Cx30- G11R (NT) and A88V (TM2) has been associated with Clouston syndrome (abnormal formation of ectodermal structures) and when they are expressed in HeLa cells induce a massive release of ATP into the extracellular medium (Essenfelder et al., 2004) and subsequent cell death (Berger et al., 2014), suggesting uncontrolled HC activity. On the other hand, the mutant Cx31R42P (which produces erythrokeratodermia variabilis) promotes an enhancement in HC activity and cell death when expressed in HeLa cells (Chi et al., 2012). It was postulated that the previous condition was mediated by an important production of free radicals as consequence of ER stress (Chi et al., 2012). Recently, a mutation in Cx43 (Cx43G8V) has been associated to keratoderma-hypotrichosis-leukonychia totalis syndrome (KHLS), a condition characterized by hyperkeratosis and alopecia, the expression of this mutant induced gain in HC activity and Ca^2+^ influx into the cells (Wang et al., 2015).

### X-linked Charcot–Marie–Tooth Disease

This neuropathy is a hereditary disease caused by different mutations in Cx32 gene (Bergoffen et al., 1993). There are several Cx32 mutations that induce Charcot–Marie–Tooth disease (Liang et al., 2005). Patients with this disease present neurodegeneration due to altered myelin production by Schwann cells (Bergoffen et al., 1993). When the mutation Cx32F235C (CT) is expressed in *Xenopus* oocytes, it induces cell death after 72 h, which was associated with changes in its voltage sensitivity (Liang et al., 2005). In addition, the other pathological mutant Cx32S85C induces a decrease in the number of HCs at the plasma membrane (measured as biotinylated protein). However, when this mutant was expressed in oocytes, larger HC currents were observed under depolarization (Abrams et al., 2002). Thus, several mutations induce this disease, but to date only two of them have been reported to form leaky HCs.

### Oculodentodigital dysplasia

It is a dominant negative inherited disorder caused by mutations in Cx43 encoding gene. ODDD’s patients exhibit abnormalities in fingers, toes, eyes, face and teeth. Mice expressing human mutations Cx43- G138R or G60S, mimic the phenotype observed in humans (Dobrowolski et al., 2008; Kozoriz et al., 2013). In the case of mice +/G60S, the area of cell death induced by brain ischemia was bigger compared to control mice; accordingly, astrocytes cultured from +/G60S mice, show enhanced HC activity (Kozoriz et al., 2013). In the other hand, mutant Cx43G138R lacks one of the typical phosphorylated forms of Cx43 (P2), and cells extracted from the +/G138R mice present increased ATP release (Dobrowolski et al., 2008). The previous results were consistent with the hypothesis that the phosphorylation state of the Cx43 CT regulates Cx43 HC activity.

### Heart Disease

Heart cells express Cx40, Cx43, and Cx45. However, their respective expression is restricted to few types of cells in the heart (Bai, 2014). For example, Cx40 is expressed only in the atria and ventricular conduction system, while Cx43 is mostly expressed in cardiomyocytes (Bai, 2014). Several Cx40 mutations have been associated with atrial-fibrillation problems, but only mutants Cx40- G38D, V85I and L211I enhance HC activity (Patel et al., 2014; Sun et al., 2014). In the case of G38D, it was found that HCs formed by this mutant present a gain of activity when N2A cells were subjected to hyperpolarization and depolarization (Patel et al., 2014). Cx43- I31M, G143S and G138R mutants (which also induce ODDD), present spontaneous arrhythmias, which were associated with both, a decrease of GJC coupling and an increase of ATP release from cardiomyocytes (Dobrowolski et al., 2007). A few years ago, it was demonstrated that down-regulation of Cx43 in cardiac fibroblasts reduce the amount of ATP released (Lu et al., 2012). The ATP released activates the pro-fibrotic response to heart insults via activation of P2Y receptors (Lu et al., 2012). Thus, increased Cx43 HC activity after -for example- myocardial infarction (John et al., 1999; Johansen et al., 2011) will lead to cardiomyocyte malfunction due to a massive entry of Ca^2+^ and Na^+^ (Li et al., 2001). In addition, it will also contribute to cardiac fibrosis (Lu et al., 2012) increasing heart failure.

## Central Nervous System Neurodegenerative Diseases

Under physiological conditions HCs participate in important functions of the nervous system (NS), as for example, in synaptic modulation (Stehberg et al., 2012; Chever et al., 2014). Moreover, it has been shown that some pathological conditions increase HC activity, in particular the activity of astrocyte HCs formed by Cx43, which have been correlated with neuronal malfunctioning and death (Orellana et al., 2012). When an ischemic episode occurs, astrocytes open their Cx43 HCs (Contreras et al., 2002; Retamal et al., 2006), probably due to dephosphorylation and *S*-nitrosylation of Cx43 (Retamal et al., 2006). The previous conditions induce a massive opening of astrocyte Cx43 HCs allowing the release of high amounts of ATP and glutamate from astrocytes (Orellana et al., 2011a; Li et al., 2015). This increment in extracellular ATP and glutamate concentration could induce the opening of neuronal Pannexin1 channels, perturbing neuron homeostasis causing cell death (Orellana et al., 2011a). Consistently, administration of Cx43 mimetic peptides, to block HCs, improved brain recovery after ischemia in fetal sheep (Davidson et al., 2012) and neonatal rats (Li et al., 2015).

Hyperactive HCs may also be involved in other brain diseases. Lysosomal storage diseases (LSDs) encompass a large group of inherited metabolic disorders characterized by the accumulation of storage material within lysosomes and HCs seems to have a relevant role in the progression of these diseases (Bosch and Kielian, 2014). In this line, an enhanced Cx43 HC activity was observed in astrocytes from a mouse model of LSD (CLN3^Δex7/8^; Finn et al., 2011; Burkovetskaya et al., 2014) which could importantly contribute to neuronal deterioration as mentioned above. On the other hand, opening of HCs could also contribute to brain deterioration in Alzheimer’s disease. Orellana et al. (2011b) reported that Aβ peptide induces massive HC opening in astrocytes, microglia, and neurons, either in culture and in hippocampal slices (Orellana et al., 2011b). This increase of HC activity is correlated with augmented release of neuroactive molecules, such as glutamate and ATP, with induction of cellular death (Orellana et al., 2011b; Bosch and Kielian, 2014). Accordingly, blockage of HCs improved memory impairment in a mouse model of Alzheimer’s disease (Takeuchi et al., 2011). Other neurodegenerative diseases in which HC have been involved are: HIV encephalitis (Eugenin and Berman, 2013; Orellana et al., 2014), amyotrophic lateral sclerosis (Boillee et al., 2006; Yamanaka et al., 2008; Takeuchi et al., 2011), Parkinson’s disease (Rufer et al., 1996; Kawasaki et al., 2009), Rasmussen encephalitis (Cepeda et al., 2015) and epilepsy (Mylvaganam et al., 2014). A common milestone of these diseases is the inflammation condition, where cytokines and reactive oxygen species (ROS) can activate HCs in glial cells (astrocytes and microglia; Retamal et al., 2007) increasing the extracellular concentration of compounds, like ATP and glutamate, that could indirectly open Pannexin1 channels leading to neuronal death (Orellana et al., 2012; Bosch and Kielian, 2014; Takeuchi and Suzumura, 2014).

## Future Directions

When opened in a controlled fashion, Cx HCs allow physiological paracrine and autocrine communication between neighboring cells. However, under certain pathological conditions, these HCs open more frequently, inducing ionic imbalance and cell lysis. In particular, specific missense mutations in Cx genes associated with human genetic disease produce leaky HCs, a condition that perturbs ionic cell homeostasis, increases ATP release and Ca^2+^ influx, which in the extreme condition leads to cell death. Probably, the major problem in the study of Cx- based channels is the lack of specific pharmacological tools able to block or open these channels. Thus, for example, one of the most used HC blockers is La^3+^ (usually used at 200 μM), but this lanthanide also blocks TRP channels (Zhao et al., 2015), cGMP-activated currents (Wang et al., 2013b) and Ca^2+^ channels (Nelson et al., 1984). Fortunately, in the last years new tools have been developed for the study of Cx- HCs. These are based on small peptides that mimic some regions of a given Cx (Iyyathurai et al., 2013). Through the use of these mimetic peptides it has been possible to study *in vitro*/*in vivo* the role of HCs in a much more specific way. Because of their specificity and high affinity, they could be used for the treatment of diseases associated with leaky HCs. In this line of thought, mimetic peptides Gap26 or Gap27 have been observed to block cardiomyocyte death induced by ischemic-like conditions *in vitro* (Shintani-Ishida et al., 2007) as well as *in vivo* (Hawat et al., 2012). In the NS, Gap26 and Gap27 peptides blocked Cx43 HC opening induced by inflammatory conditions (Retamal et al., 2007; Froger et al., 2010), while Gap27 reduced spontaneous epileptiform activity in organotypic hippocampal slice cultures and cell death associated with this activity (Samoilova et al., 2008; Yoon et al., 2010). On the other hand, mimetic peptide Gap26 inhibits the spread of damage from the trauma zone to the penumbra in an *in vitro* model (Rovegno et al., 2015). Similar results have been observed *in vivo* in a model of spinal cord injury (Huang et al., 2008; O’Carroll et al., 2008) and post-ischemic brain injury (Davidson et al., 2012). Moreover, inhibition of Cx43 HCs with mimetic peptides in the spinal cord, significantly reduced the sensitization to neuropathic pain (Chen et al., 2014), which suggests that opening of HCs could result in an excessive release of neuroactive molecules in chronic pain. Accordingly, exposure of sensory ganglia to mimetic peptides, to block Cx43 HCs of satellite glial cells, reduced sensory neuron activity (Retamal et al., 2014a,b). Therefore, mimetic peptides could be used as the starting point to develop new and more specific pharmacologic agents to inhibit HCs to treat human diseases associated to leaky HCs.

## Conflict of Interest Statement

The authors declare that the research was conducted in the absence of any commercial or financial relationships that could be construed as a potential conflict of interest.
